# Supplementary material for: Integration of an OS-Based Machine Learning Score (AS Score) and Immunoscore as Ancillary Tools for Predicting Immunotherapy Response in Sarcomas
Source: Cancers (Basel). 2025 Aug 1;17(15):2551. doi: 10.3390/cancers17152551 (PMC12346403; doi:10.3390/cancers17152551)
Supplement: Supplementary file 1 [file cancers-17-02551-s001.zip › cancers-3724272-Supplementary.pdf]

Supplementary tables

Supplementary Table S1: Main clinicopathological characteristics of the TCGA sarcoma cohort (n=253)

PATIENT CHARACTERISTICS	
**Age at diagnosis (years)**	
**Median**	61
**Range**	20-90
**Gender**	
**Male**	116 (45.8)
**Female**	137 (54.2)
**FOLLOW-UP AND OUTCOMES**	
**Overall survival**	
**N**	253
**Events**	75
**Median (range) months**	18.33 (0-117.47)

TUMOR CHARACTERISTICS	
**Tumor size (cm)**	
**Median**	10.15
**Range**	0.5-39.5
**Mitotic rate**	
**Median**	17
**Range**	0-102
**Margins**	
**Negative**	137 (54.15)
**Positive**	74 (29.25)
**Unknown**	42 (16.6)
**Surgery**	
**Yes**	253 (100)
**No**	0 (0)
**Radiotherapy treatment**	
**Yes**	35
**No**	176

Supplementary Table S2: The 10 most significant genes evaluated by the maxstat algorithm.

Gene	Cut-off	p-value	OS p-value
SERPINE1	13.66	0.058	1.54*10-6
CD200	12.28	0.011	7.05*10-4
HES1	13.34	0.001	8.47*10-4
SMAD2	13.55	0.003	1.17*10-3
MT2A	16.27	0.212	1.30*10-3
CCND1	15.33	0.014	1.36*10-3
IGF1R	12.75	0.001	1.64*10-3
PDK2	11.55	0.019	1.70*10-3
TCF12	13.82	0.002	1.77*10-3
MAPK1	13.25	0.015	1.81*10-3

Supplementary Table S3: Significant Gene-performance in cox regression analysis.

**Gene**	**HR**	**p-value**	**weight**
SERPINE1	2.32	<0.001	1.00977
IGF1R	0.32	0.001	–1.22281
TCF12	1.78	0.133	0.61161
MAPK1	0.44	0.026	–0.63790

Supplementary Table S4: Univariate and multivariate Cox regression analysis of clinicopathological parameters and AS score in the angiosarcoma cohort. *The analysis was performed based on the available clinical data.

	**Univariate**					**Multivariate**			
**Variable**	**Levels**	**N**	**Events**	**Median (months)**	**p-value**	**HR**	**CI**	**p-value**	**global p-value**
**Primary site**	Cutaneous	17	12	24	0.0012	N.S.	N.S.	N.S.	0.00017
	Non-cutaneous visceral	6	6	9					
	Non-cutaneous soft tissue	2	2	10					
**Age**	Age median	12	10	11	0.68	N.S.	N.S.	N.S.	
	Age median	13	10	20					
**Gender**	Female	14	12	19	0.71	N.S.	N.S.	N.S.	
	Male	11	8	15					
**Surgery**	Yes	22	18	19	0.36	N.S.	N.S.	N.S.	
	No	3	2	13.5					
	Yes	9	9	9	0.0003		N.S.	N.S.	

**Chemotherapy**	No	16	11	24		N.S.			
**Margins**	afectos	4	4	6	0.0049	N.S.	N.S.	N.S.	
	libres	21	16	20					
**Mitotic rate**	<med	8	5	19	0.7	N.S.	N.S.	N.S.	
	>med	9	7	12					
**Tumor size**	<med	9	7	10	0.89	N.S.	N.S.	N.S.	
	>med	13	10	19					
**AS score**	Low	11	7	20	0.0001	7	2.4-21	0.0004	
	High	14	13	7					

Supplementary Table S5: Univariate and multivariate Cox regression analysis of clinicopathological parameters, AS score, and Immunoscore in the TCGA sarcoma cohort. *The analysis was conducted using all available clinical information.

	**Univariate**					**Multivariate**			
**Variable**	**Levels**	**N**	**Events**	**Median (days)**	**p-value**	**HR**	**CI**	**p-value**	**global p-value**
**Age**	Age median	125	31	2034	0.06	N.S.	N.S.	N.S.	$9.942*10^{-5}$
	Age median	128	44	1424					
**Gender**	Female	137	41	1953	0.9	N.S.	N.S.	N.S.	
	Male	116	34	1825					
**Radiotherapy**	Yes	176	49	1953	0.3	N.S.	N.S.	N.S.	
	No	35	13	1366					
**Margins**	negative	131	30	2448	0.04	N.S.	N.S.	N.S.	
	positive	72	23	1627					
**Mitotic rate**	<med	21	2	2448	0.04	N.S.	N.S.	N.S.	
	>med	65	17	1478					
**Tumor size**	<med	122	27	2464	0.003	N.S.	N.S.	N.S.	
	>med	122	46	1424					
**AS score**	Low	206	58	1953	0.007	2.47	1.42-4.3	0.001420	
	High	47	17	1175					
**IS score**	Low	153	55	1262	0.0023	0.42	0.25-0.7	0.000902	
	High	100	20	2034					
